# Shared circulating diagnostic biomarkers and molecular mechanisms in ischemic stroke and systemic lupus erythematosus

**DOI:** 10.3389/fimmu.2025.1565379

**Published:** 2025-04-17

**Authors:** Xiaoyi Ma, Lifei Huang, Huanhuan Yan

**Affiliations:** ^1^ Department of Geriatrics, The First Affiliated Hospital, Sun Yat-Sen University, Guangzhou, China; ^2^ MRI Division, Wuhan United Imaging Life Science Instrument Co., Ltd., Wuhan, China; ^3^ Brain Science Laboratory, Shenzhen United Imaging Research Institute of Innovative Medical Equipment, Shenzhen, China; ^4^ Paul C. Lauterbur Research Center for Biomedical Imaging, Shenzhen Institutes of Advanced Technology, Chinese Academy of Sciences, Shenzhen, China

**Keywords:** ischemic stroke, systemic lupus erythematosus, diagnostic biomarkers, bioinformatics, immune infiltration

## Abstract

**Introduction:**

Ischemic stroke, a prevalent cerebrovascular disorder characterized by reduced cerebral blood flow, and systemic lupus erythematosus (SLE), an autoimmune disease affecting various organs, are suspected to share overlapping etiological mechanisms and genetic predispositions. This study aimed to identify shared diagnostic biomarkers and molecular mechanisms by analyzing datasets from the GEO database.

**Methods:**

We pinpointed differentially expressed genes using the limma package and identified co-expression modules associated with both conditions using Weighted Gene Coexpression Network Analysis. Pathway enrichment analysis was conducted using GO and KEGG to identify co-driver genes. LASSO regression was applied to evaluate potential diagnostic markers, and immune cell infiltration was quantified using the CIBERSORT computational method. A middle cerebral artery occlusion (MCAO) mouse model was developed to assess core gene expression *in vivo*.

**Results:**

We identified 69 shared driver genes linked to stroke and SLE, which were narrowed down to the top 10 genes through a Protein-Protein Interaction network analysis with Cytoscape. LASSO regression selected EIF2AK2, PARP9, and IFI27 as diagnostic biomarkers, supported by ROC curve analysis. Immune cell infiltration profiles were nearly identical between ischemic stroke and SLE. 9.4T MR imaging, H&E and Nissl staining confirmed ischemic stroke in the MCAO model, and qPCR analysis confirmed elevated expression of the three hub genes.

**Discussion:**

Our findings provide evidence for common diagnostic indicators and disease mechanisms in ischemic stroke and SLE, offering novel insights for potential therapeutic strategies targeting their shared immune cell infiltration microenvironments.

## 1 Introduction

Ischemic stroke, a leading cause of global mortality and disability, affects millions of individuals annually (1). Characterized by a sudden loss of cerebral function due to the disruption of blood flow, whether through ischemia or hemorrhage, stroke has profound public health implications, with significant morbidity and mortality rates. According to data from the World Stroke Organization (WSO), stroke ranks as the number two cause of mortality worldwide and is the third most frequent contributor to the combined metric of death and disability. Between 1990 and 2019, there was a significant increase in the burden of stroke, with a 70% increase in incident stroke cases, a 43% increase in stroke-related deaths, a 102% increase in stroke prevalence (2, 3). Genetic and molecular research has identified several key proteins and genes associated with stroke pathology. Notably, matrix metalloproteinases (MMPs) are implicated in the degradation of the blood-brain barrier, contributing to subsequent brain edema (4, 5). Furthermore, the exacerbating role of inflammatory cytokines such as IL-6 and TNF-α in stroke outcomes is well-established (6). These molecules, which are involved in neuronal damage, represent potential therapeutic targets. Elucidating the molecular mechanisms of stroke is crucial for advancing diagnostic and therapeutic strategies.

Systemic Lupus Erythematosus (SLE) is a chronic autoimmune disorder that mainly affects women and manifests a spectrum of clinical symptoms (7). Characterized by the production of autoantibodies targeting nuclear and cytoplasmic antigens, SLE induces widespread inflammation and tissue damage. This disease impacts multiple organ systems, including the skin, kidneys, joints, and central nervous system. SLE’s prevalence varies globally, with higher incidences in populations of African, Asian, and Hispanic descent (8). Genetic studies have identified susceptibility genes, such as those encoding HLA-DR and complement components, which are integral to SLE’s pathogenesis (9). Research has also underscored the role of cytokines like IFN-α and IL-10 in modulating immune responses in SLE patients (10). These insights are pivotal for developing targeted therapies aimed at immune system modulation and disease activity reduction in SLE patients.

SLE samples are distinguished by the presence of autoantibodies and immune complexes that exacerbate inflammation and tissue damage. In the context of stroke, these autoantibodies can intensify the inflammatory response, worsening clinical outcomes (7). Studies indicate that SLE patients face an elevated risk of stroke, particularly ischemic stroke, due to antiphospholipid antibodies and other pro-thrombotic factors (11). Conversely, stroke can incite an autoimmune response resembling SLE, posing diagnostic challenges and complicating patient management (12). The interplay between stroke and SLE highlights the necessity of comprehending the underlying molecular mechanisms and identifying biomarkers to facilitate diagnosis and management of these conditions.

Stroke samples display distinct molecular and cellular alterations reflective of the disease’s pathophysiology. The ischemic insult triggers a cascade of events, including excitotoxicity, oxidative stress, and inflammation, culminating in neuronal death and brain damage (13, 14). Immune cells, such as microglia and infiltrating leukocytes, are instrumental in mediating the post-stroke inflammatory response in the brain (15). The identification of specific biomarkers and molecular signatures in stroke samples can offer invaluable insights into the disease process and guide the development of targeted therapies. Moreover, comparing molecular changes between stroke and SLE samples may reveal shared pathways and potential therapeutic targets beneficial for patients with either condition.

This study endeavors to identify diagnostic biomarkers for stroke and SLE through bioinformatics methodologies, focusing on immune infiltration analysis and candidate drug identification. Gene expression profiles will be leveraged to identify genes with altered expression and to map out networks of protein-protein interactions (PPI). Machine learning techniques, including LASSO, will aid in the selection of key biomarkers, while the CIBERSORT algorithm will assess immune cell proportions. Additionally, the DGIdb platform will be employed to identify candidate drugs targeting hub genes.

To further validate our findings, the expression of the core genes will be verified in an ischemic stroke mouse model. The insights gleaned from this study may significantly contribute to understanding the molecular underpinnings of stroke and SLE, thereby informing the creation of innovative diagnostic and therapeutic approaches.

## 2 Methods

### 2.1 Transcriptome data preprocessing

**Figure 1:**
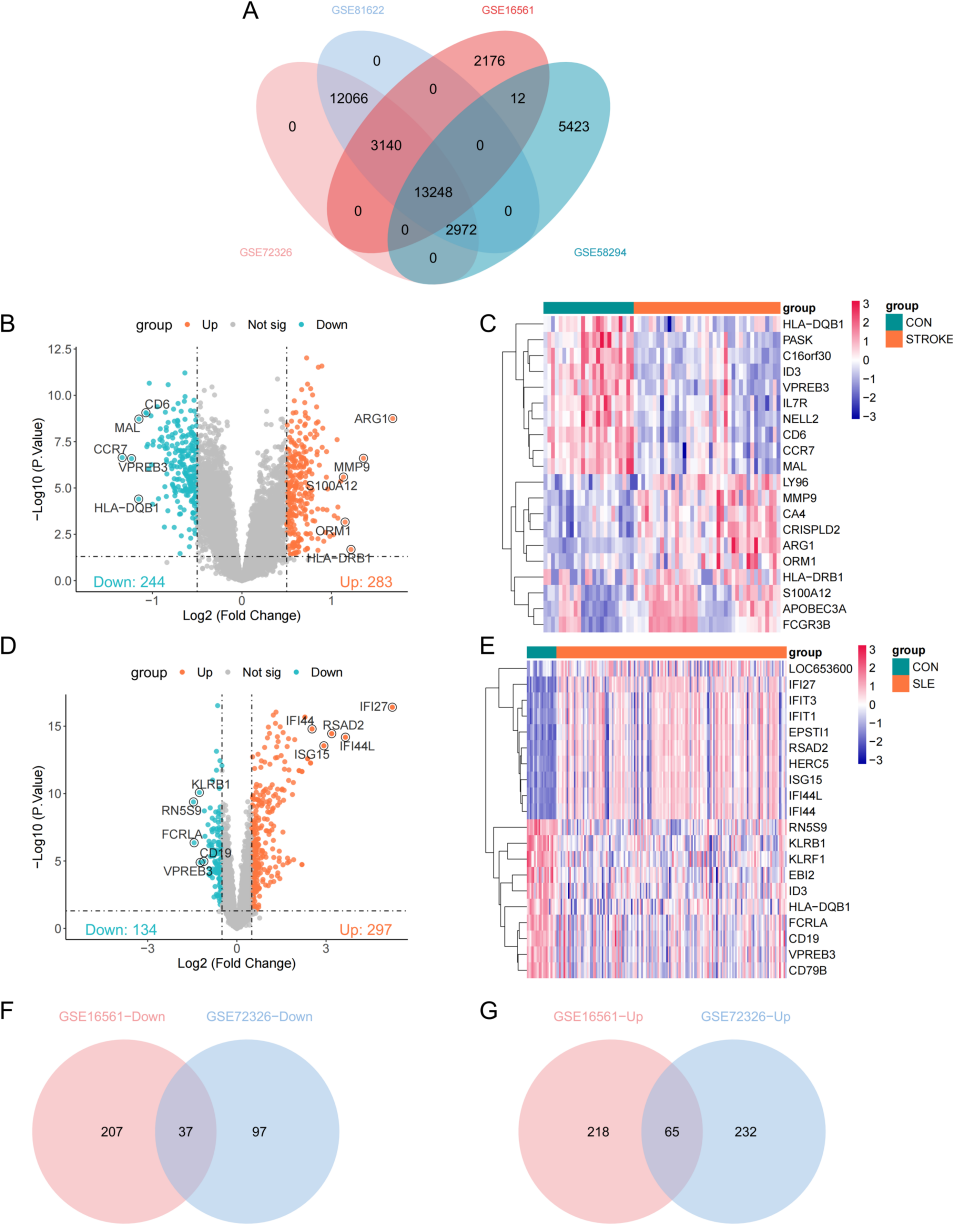
Identification of differentially expressed genes (DEGs). **(A)** Venn plots of crossover genes for the four cohort sets. **(B)** Volcano plot showing the distribution of DEGs in stroke-GSE16561. Ching color represents down-regulated genes and orange color represents up-regulated genes. **(C)** Heatmap of the top 10 genes with the most prominent differential expression in stroke-GSE16561 cohort. **(D, E)** Volcano plot and heatmap of SLE-GSE72326 cohort. **(F, G)** Venn diagram of overlapping down-DEGs and up-DEGs in stroke and SLE.

Consistent with methodologies from previous studies, peripheral blood transcriptome datasets GSE16561, GSE58294, GSE72326, and GSE81622 were examined in this study (16, 17). In the context of ischemic stroke, we incorporated 39 stroke cases and 24 controls from GSE16561, along with 69 stroke cases and 23 controls from GSE58294. For SLE, the analysis encompassed 157 SLE cases and 20 controls from GSE72326, complemented by 15 SLE cases and 25 controls from GSE81622. These datasets encompassed peripheral blood mononuclear cells (PBMCs). We performed GeneSymbol mapping for the transcriptome data according to the respective platforms, selecting the median value in instances of multiple matches. The definitive expression matrix was compiled after applying the log2(X+1) normalization method. After the preliminary quality control step, we employed the ‘normalizeBetweenArrays’ function from the ‘limma’ package to perform quantile normalization. This process aligns the empirical distribution of expression values across all samples, effectively minimizing technical disparities. Subsequently, we focused on a subset of 13,248 genes that were consistently present in all 4 datasets for further bioinformatics analysis (**Figure 1A**).

### 2.2 Selection of potential diagnostic markers

We performed an analysis of gene expression differences across the GSE16561 and GSE72326 datasets, leveraging the limma package with a significance threshold of p value < 0.05 and a fold change magnitude of |LogFC| > 0.5. To address the issue of multiple comparisons, we applied the Benjamini-Hochberg method for false discovery rate control. In the Weighted Gene Coexpression Network Analysis (WGCNA) (18), an input matrix was constructed using all genes from the GSE16561 and GSE72326 datasets. Topological calculations were conducted with soft thresholding from 1 to 20 to determine the optimal soft threshold. The correlation matrix was first converted into an adjacency matrix, and then this adjacency matrix was further transformed into a topological overlap matrix (TOM). Clusters of modules were determined through the application of average linkage hierarchical clustering, which was based on the TOM. Modules exhibiting similarity were consolidated. The Pearson correlation coefficient was utilized to pinpoint the modules that exhibited the most robust positive and negative correlations with the disease phenotype, designating these as the core modules. Gene significance (GS) was quantified by the correlation between individual gene expressions and the disease trait, while module membership (MM) was ascertained by the alignment of gene expression patterns with the principal component of their respective module.

### 2.3 Gene ontology and pathway enrichment analysis

Utilizing the clusterProfiler package, we performed enrichment analyses to explore the functional annotations within the Gene Ontology (GO) framework and to identify significant pathways through the Kyoto Encyclopedia of Genes and Genomes (KEGG) on the identified common driver genes. GO terms were assigned to elucidate the biological processes (BP), molecular functions (MF), and cellular components (CC) associated with the genes, whereas KEGG was employed for pathway-based annotation. Significance in enrichment was set at a threshold of P < 0.05.

### 2.4 Construction of PPI networks

We integrated a cohort of 69 potential shared driver genes into the STRING database (https://cn.string-db.org/), omitting any orphan genes. Subsequently, Cytoscape was employed to discern pivotal genes and to construct a graphical representation of the network. Within Cytoscape, the aforementioned genes were subjected to analysis to determine the top 10 genes within the PPI network, employing the Maximal Clique Centrality (MCC) algorithm for this calculation.

### 2.5 Machine learning for diagnostic marker identification

We employed the Least Absolute Shrinkage and Selection Operator (LASSO) regression technique to pinpoint crucial genes. This method incorporates a penalty term that drives certain regression coefficients to zero, simplifying the model and alleviating the effects of multicollinearity (19). The top ten genes, as determined by the MCC algorithm, were inputted into the LASSO model based on their expression profiles, with the occurrence of disease as the binary outcome variable for the selection of diagnostic biomarkers.

### 2.6 Analysis of immune cell infiltration

We employed the CIBERSORT algorithm to deduce the relative abundance of diverse immune cell subtypes from the gene expression profiles associated with immune cell-specific genes (20). The resulting data for 22 distinct immune cell infiltrates were consolidated into a comprehensive matrix for further analysis. Moreover, the Spearman correlation method was applied to evaluate the relationship between the key biomarkers and the expression levels of the infiltrating immune cells, with statistical significance adjustments made using the Benjamini-Hochberg procedure for multiple comparisons.

### 2.7 Gene Set Enrichment Analysis

Gene Set Enrichment Analysis (GSEA) was conducted using the enrichR package in R software (version 4.3.3; R Foundation for Statistical Computing, Vienna, Austria). Gene expression data underwent pre-processing and normalization. Samples were categorized into low and high expression groups based on the bottom and up 25% of expression levels, respectively. Subsequently, the GSEA algorithm was applied to determine enrichment scores and corresponding p-values for each gene set under investigation.

### 2.8 Drug candidate selection

The shared central genes associated with both stroke and SLE were submitted to the DGIdb database for analysis (https://www.dgidb.org/) (21, 22). Drug candidates associated with these shared hub genes were then identified.

### 2.9 Animal experiments

Middle cerebral artery occlusion (MCAO) was induced in 3-month-old male C57BL/6 mice using a modified method (23). Mice were anesthetized with 3% isoflurane for induction, maintained at 1.5%, and core temperature was kept at 37 ± 0.5°C via a heating system. A midline neck incision exposed the external carotid artery (ECA), which was dissected from the vagus nerve. A 7-0 nylon suture, coated with silicone, was inserted into the ECA and advanced to the internal carotid artery (ICA) to occlude the MCA. Occlusion was verified by a >80% drop in cerebral blood flow, measured by laser Doppler flowmetry (PeriFlux System 5000). The occlusion lasted 50 minutes before the suture was removed. Arterial blood gases were monitored to maintain physiological levels (PO2: 120 ± 10 mmHg; PCO2: 35 ± 3 mmHg). After surgery, the mice were monitored until they recovered. The study was approved by the Experimental Animal Ethics Committee of Zhongshan School of Medicine, Sun Yat-sen University, Guangzhou, China. Mice (22-26 grams) were from the Animal Center of Sun Yat-Sen University.

### 2.10 MRI imaging

Magnetic resonance imaging (MRI) was performed on a 9.4 Tesla magnet with a 30-cm bore diameter (uMR 9.4T, United Imaging Life Science Instruments, Wuhan, China), featuring a gradient system capable of generating up to 1000mT/m in any direction. An 86 mm quadrature resonator was utilized for transmission, and a three-channel mouse brain surface coil (Mouse Brain Surface Coil-3) was employed for signal reception. Mice (n = 4) were administered anesthesia through a mixture of 2% isoflurane in air, inducing gaseous anesthesia. Subsequently, they were placed on an MRI-compatible animal bed. Throughout the procedure, the respiratory rate of the animals was closely monitored. A circulating warm water system was employed to maintain body temperature at a stable 36.5 ± 0.5 °C. The acquired MRI sequence parameters were as follows: 2D Multi echo spin echo sequences: Repetition Time = 2000ms, TE1/delta TE/TEn7 = 7.34/7.34/51.38 ms, Number of slices = 15, Thick of slices = 0.5mm, Readout FOV = 20mm, Phase-Encoding FOV = 18mm, Bandwidth = 250Hz, Matrix = 208 (RO) * 187(PE), Averages = 4. Data analysis was conducted using the U_VIEWER software (R001, Shanghai United Imaging Healthcare Co., Ltd.). Subsequently, the MRI data collected were utilized for statistical evaluation.

### 2.11 H&E and Nissl staining

At 24 hours post-MCAO, the mice (n = 4) were euthanized, and their brains were harvested. Following the procedure, *in situ* cardiac perfusion was promptly performed using a 4% paraformaldehyde (PFA) solution in phosphate-buffered saline (PBS) to ensure thorough tissue fixation. Post perfusion, the brains were extracted and further fixed in PFA for a duration of 24 hours at 4°C to maintain tissue integrity. For Hematoxylin and Eosin (H&E) staining, the tissue sections were subjected to deparaffinization and rehydration. They were then stained with Harris hematoxylin, differentiated in a 0.5% hydrochloric acid solution, counterstained with eosin Y, dehydrated, and finally coverslipped. Nissl staining involved staining sections with 0.1% cresyl violet acetate, rinsing, dehydrating, and coverslipping. Both stained sections were examined under a light microscope to evaluate tissue morphology and neuronal characteristics.

### 2.12 Quantitative real-time PCR

RNA extraction and quantitative real-time PCR (qPCR) were performed according to previously described methods (24). The primer sequences used for qPCR are summarized in **Table 1**. Gene expression levels were normalized to β-actin and presented as 2^-ΔΔCT^.

**Table 1:** Primer sequences for qPCR.

Gene	Forward Primer Sequence	Reverse Primer Sequence
TNF-α	5′-CTTGTTGCCTCCTCTTTTGCTTA-3′	5′-CTTTATTTCTCTCAATGACCCGTAG-3′
IL-6	5′-TCACAGAAGGAGTGGCTAAGGACC-3′	5′-ACGCACTAGGTTTGCCGAGTAGAT-3′
IL-1β	5′-ATTGTGGCTGTGGAGAAG-3′	5′-AAGATGAAGGAAAAGAAGGTG-3′
EIF2AK2	5′-ATGCACGGAGTAGCCATTACG-3′	5′-TGACAATCCACCTTGTTTTCGT-3′
PARP9	5′-AGGACGCCAAAGGGATCTG-3′	5′-CCGGCTCCATAAACTGGGT-3′
IFI27	5′-GCTTGTTGGGAACCCTGTTTG-3′	5′-GGATGGCATTTGTTGATGTGGAG-3′
β-actin	5′-TGTCCACCTTCCAGCAGAT-3′	5′-CTCAGTAACAGTCCGCCTAGA-3′

### 2.13 Statistical analysis

Quantitative outcomes are reported as the mean ± standard deviation (SD) for each experimental group. Data analysis and visualization were performed using GraphPad Prism software (version 8, GraphPad Software, La Jolla, CA, USA). Statistical significance was determined with a p-value threshold of less than 0.05 (p < 0.05). The levels of statistical significance for the experimental results are indicated by the following notations: *p < 0.05, **p < 0.01, ***p < 0.001, and ****p < 0.0001.

## 3 Results

### 3.1 Identification of DEGs in stroke and SLE

An initial identification of 527 differentially expressed genes (DEGs) was conducted based on the stroke dataset (GSE16561), including 283 upregulated genes and 244 downregulated genes (**Figure 1B**). A volcano plot was used to visualize these DEGs, while a heatmap was utilized to present the top 10 significantly upregulated and downregulated genes among these DEGs (**Figures 1B, C**). Notably, ARG1 emerged as the most significantly upregulated gene in stroke samples (**Figure 1B**). Moreover, 431 DEGs were discovered from the SLE dataset (GSE72326), comprising 297 upregulated genes and 134 downregulated genes, with IFI27 identified as the most significantly upregulated gene in SLE samples (**Figures 1D, E**). Subsequently, through intersection analysis, 37 DEGs were found to be commonly downregulated and 65 DEGs were commonly upregulated across both datasets (**Figures 1F, G**).

### 3.2 WGCNA of stroke and SLE

**Figure 2:**
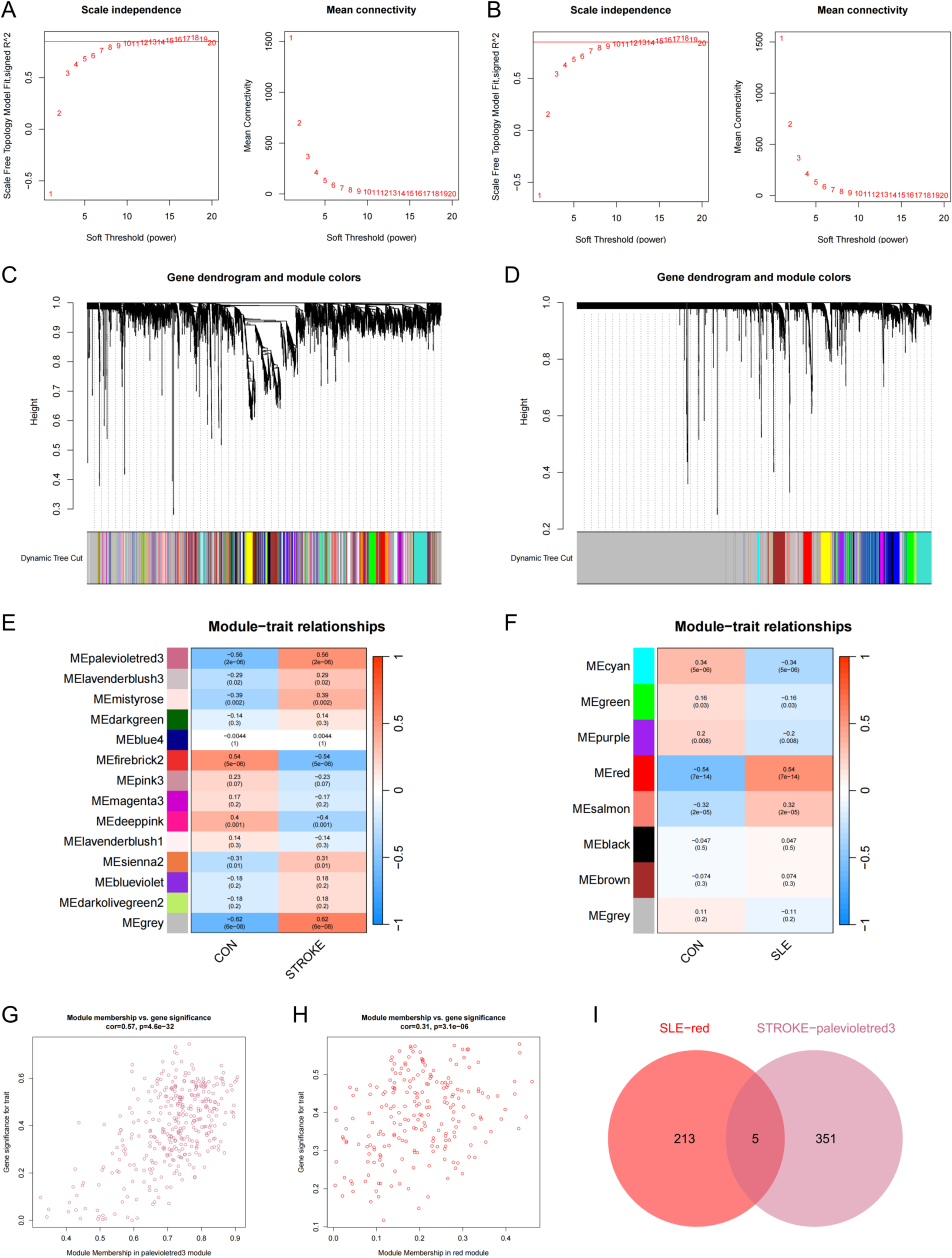
WCGNA analysis of stroke and SLE. **(A, B)** Mean connectivity for scale independence and soft threshold (β) in the stroke-GSE16561 cohort and the SLE-GSE72326 cohort. **(C, D)** Clustering dendrograms of genes in stroke and SLE. **(E, F)** Heatmap of the correlation analysis of module genes with clinical phenotypes in stroke and SLE. Red color represents positive correlation and blue color represents negative correlation. **(G)** Association between gene significance (GS) and module membership (MM) within the palevioletred3 module of stroke. **(H)** Association between GS and MM within the red module of SLE. **(I)** Venn diagram for intersecting genes between palevioletred module in stroke and red module in SLE.

Subsequently, WGCNA was executed on the stroke dataset GSE16561 and the SLE dataset GSE72326 to investigate the correlation between clinical traits and genes. No notable outlier samples were detected in either the SLE or stroke datasets. Utilizing the WGCNA method, the optimal soft threshold was determined to be 15 for both stroke and SLE datasets (**Figures 2A, B**). Through module similarity assessment, 14 modules were identified in the stroke dataset and 8 modules in the SLE dataset (**Figures 2C, D**). Analyzing the relationship between genetic modules and specific conditions revealed that the palevioletred3 module had the highest positive correlation with stroke, with a correlation coefficient of 0.56 as depicted in **Figure 2E**. Similarly, the red module showed the most significant positive correlation with SLE, with a coefficient of 0.54, as illustrated in **Figure 2F**. Notably, a strong link was identified between gene significance (GS) and module membership (MM) across the modules, with respective correlation coefficients of 0.57 for stroke and 0.31 for SLE (**Figures 2G, H**), indicating a significant relationship between module genes and disease occurrence. Ultimately, WGCNA identified 5 overlapping genes that may play a key role in the development of stroke and SLE (**Figure 2I**).

### 3.3 Enrichment analysis of common driver genes in stroke and SLE

**Figure 3:**
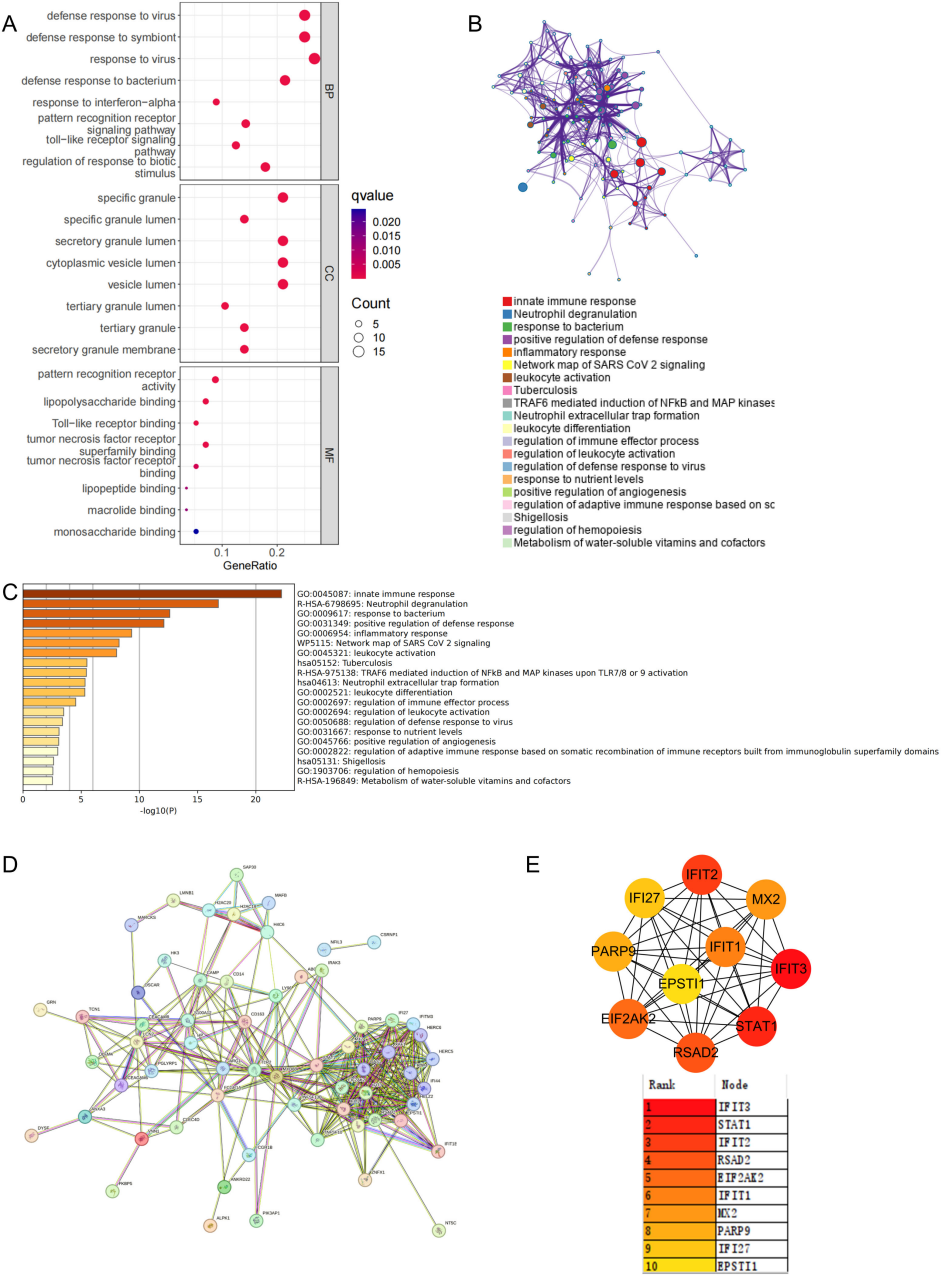
Functional enrichment and pathway enrichment of stroke and SLE co-driver genes. **(A)** GO analysis and KEGG analysis of 69 candidate common driver genes. **(B, C)** Enrichment analysis of 69 candidate common driver genes using Metascape online tool. **(D, E)** PPI network analysis of top 10 common driver genes.

In the previous analysis, 65 commonly elevated DEGs were identified in stroke and SLE, and 5 overlapping genes were found in the stroke and SLE modules. Given that the gene modules identified through WGCNA represent a subset of genes with correlated expression patterns, and may not encompass all DEGs, particularly those pivotal for disease advancement, we decided to merge the 65 DEGs with the genes from the 5 modules for further analysis. By removing duplicate genes, we obtained 69 candidate genes that may play a key role in the molecular mechanisms regulating stroke and SLE. Consequently, our initial step was to conduct GO and KEGG enrichment analyses on the selected genes. The findings indicated that these genes participate in cytokine-cytokine receptor interactions, pathways that modulate immune responses, and other pathways (**Figure 3A**).

Furthermore, to clarify the enriched pathways for the peripheral circulation marker-associated genes mentioned earlier, our analysis in the Metascape database showed that these genes were categorized into distinct functional groups. Notably, the most significant were those involved in the positive regulation of innate immune responses and neutrophil-mediated inflammatory reactions (**Figure 3B**). Concurrently, the enrichment analysis utilizing the Metascape database highlighted a shared involvement of immune and inflammatory processes in the pathogenesis of stroke and SLE, as depicted in **Figure 3C**. Subsequently, to refine our selection of genes within the same functional group, we imported the 69 potential driver genes into the STRING database, excluding those that were not interconnected (**Figure 3D**). Following this, the MCC algorithm within Cytoscape was employed to identify the top 10 genes from the PPI network, focusing on the genes previously discussed. Ultimately, IFIT3, STAT1, IFIT2, RSAD2, EIF2AK2, IFIT1, MX2, PARP9, IFI27, and EPSTI1 were designated as potential diagnostic biomarkers, with IFIT3 emerging as the most prominent among them (**Figure 3E**).

### 3.4 Identification and validation of potential shared hub genes by LASSO

**Figure 4:**
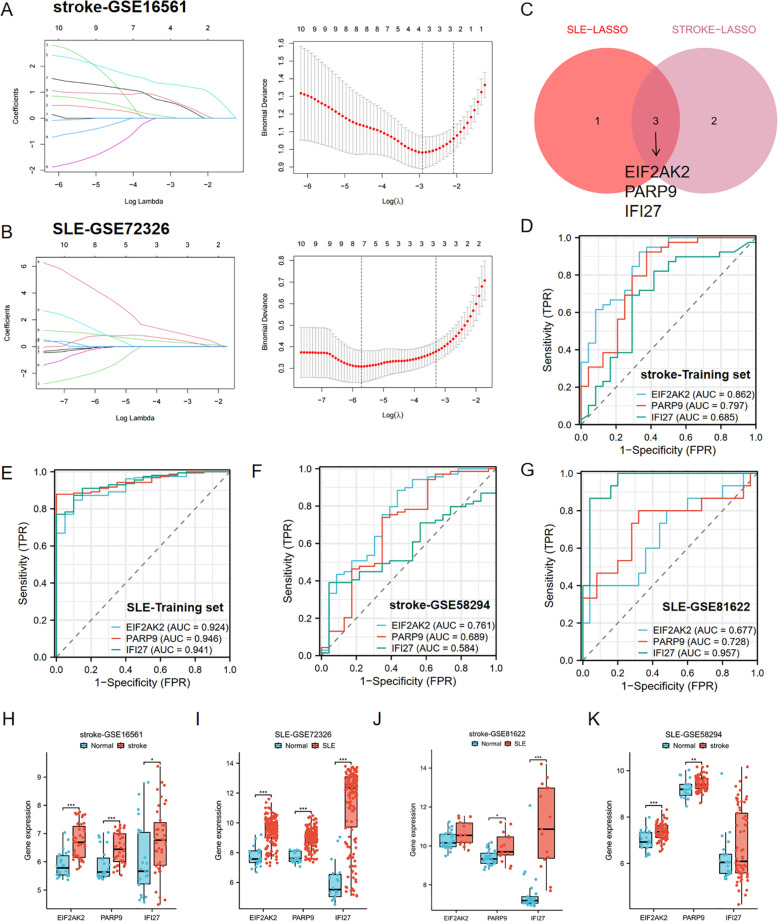
Identification of hub genes by LASSO and ROC analysis. **(A, B)** LASSO regression analysis of the stroke-GSE16561 cohort and the SLE-GSE72326 cohort. **(C)** Cross-identification of optimal hub genes using LASSO. **(D)** ROC curves for three shared diagnostic markers in the stroke-GSE16561 cohort. **(E)** ROC curves for three shared diagnostic markers in the SLE-GSE72326 cohort. **(F)** ROC curves for three shared diagnostic markers in the stroke-GSE58294 cohort. **(G)** ROC curves for three shared diagnostic markers in the SLE-GSE81622 cohort. **(H, I)** Expression of three hub genes in stroke and SLE training set (stroke-GSE16561 and SLE-GSE72326). **(J, K)** Expression of three hub genes in stroke and SLE testing set (stroke-GSE58294 and SLE-GSE81622). *p< 0.05, **p< 0.01, ***p< 0.001.

In order to identify key genes with the highest diagnostic value, we performed further LASSO regression analysis on the aforementioned 10 candidate genes using machine learning algorithms. The LASSO method selected 4 genes from the SLE dataset and 5 genes from the stroke dataset (**Figures 4A, B**). By taking the intersection of the selected genes, we ultimately identified 3 key genes with the highest diagnostic value, this is EIF2AK2, PARP9, and IFI27 (**Figure 4C**).

Furthermore, the diagnostic predictive value of the aforementioned 3 key genes was assessed using ROC curves (**Figures 4D–G**). In the stroke-GSE16561 dataset, the AUC values for EIF2AK2, PARP9, and IFI27 were 0.862, 0.797, and 0.685, respectively, all exceeding 0.6 (**Figure 4D**). In the SLE-GSE72326 dataset, the AUC values for EIF2AK2, PARP9, and IFI27 were 0.924, 0.946, and 0.941, respectively, all greater than 0.9 (**Figure 4E**). The findings suggest that these trio of genes demonstrates strong diagnostic capabilities and could be utilized as biomarkers for the identification of stroke and SLE.

The AUC values across various cohorts within the validation set demonstrated robust predictive accuracy. In the stroke validation set (GSE58294), the AUC values for EIF2AK2, PARP9, and IFI27 were 0.761, 0.689, and 0.584, respectively (**Figure 4F**). Moreover, in the SLE validation set (GSE81622), the AUC values for EIF2AK2, PARP9, and IFI27 were 0.677, 0.728, and 0.957, respectively (**Figures 4H, I**). The boxplot results showed that these three diagnostic markers were significantly upregulated in the disease group in both the stroke and SLE training sets (**Figures 4H, I**). More importantly, consistent differential trends were observed in the stroke and SLE validation sets (**Figures 4J, K**).

### 3.5 Drug candidates identification based on hub genes

Utilizing the DGIdb database (https://www.dgidb.org/), we calculated the predicted small molecule compounds for EIF2AK2, PARP9, and IFI27 individually, and identified the intersection of these compounds. This analysis yielded 28 common compounds (**Supplementary Figure 1**). Among these, 1,2-Dimethylhydrazine, Carbon Tetrachloride, Cellulose, Perfluorooctane Sulfonic Acid, Sodium Arsenite, and Vancomycin were found to downregulate the expression of EIF2AK2, PARP9, and IFI27. Conversely, Alpha-Chlorohydrin, Estradiol, Oxaliplatin, S-(1,2-Dichlorovinyl)Cysteine, Temozolomide, Topotecan, and Tretinoin were identified as compounds that upregulate the expression of these key genes (**Table 2**).

**Table 2:** Identification of candidate drugs based on key genes.

Chemical Name	Interaction Actions with EIF2AI2	Interaction Actions with PARP9	Interaction Actions with IFI27
alpha-Chlorohydrin	increases	increases	increases
Estradiol	increases	increases	increases
Oxaliplatin	increases	increases	increases
S-(1,2-dichlorovinyl)cysteine	increases	increases	increases
Temozolomide	increases	increases	increases
Topotecan	increases	increases	increases
Tretinoin	increases	increases	increases
1,2-Dimethylhydrazine	decreases	decreases	decreases
Carbon Tetrachloride	decreases	decreases	decreases
Cellulose	decreases	decreases	decreases
perfluorooctane sulfonic acid	decreases	decreases	decreases
sodium arsenite	decreases	decreases	decreases
Vancomycin	decreases	decreases	decreases
bisphenol A	increases	decreases	affects expression
Cadmium Chloride	increases	increases	decreases
Lipopolysaccharides	increases	affects reaction	increases
Plant Extracts	increases	affects expression	increases
Tetrachlorodibenzodioxin	increases	affects expression	increases
titanium dioxide	increases	increases	affects expression
Air Pollutants	decreases	increases	increases
Benzo(a)pyrene	decreases	affects methylation	increases
cobaltous chloride	decreases	decreases	increases
Cyclosporine	decreases	increases	increases
Doxorubicin	decreases	increases	decreases
Particulate Matter	decreases	increases	decreases
Acetaminophen	affects expression	decreases	decreases
Tamoxifen	affects expression	affects expression	affects expression
Valproic Acid	affects expression	affects expression	decreases

### 3.6 Immune cell infiltration analysis and its link to central genes

**Figure 5:**
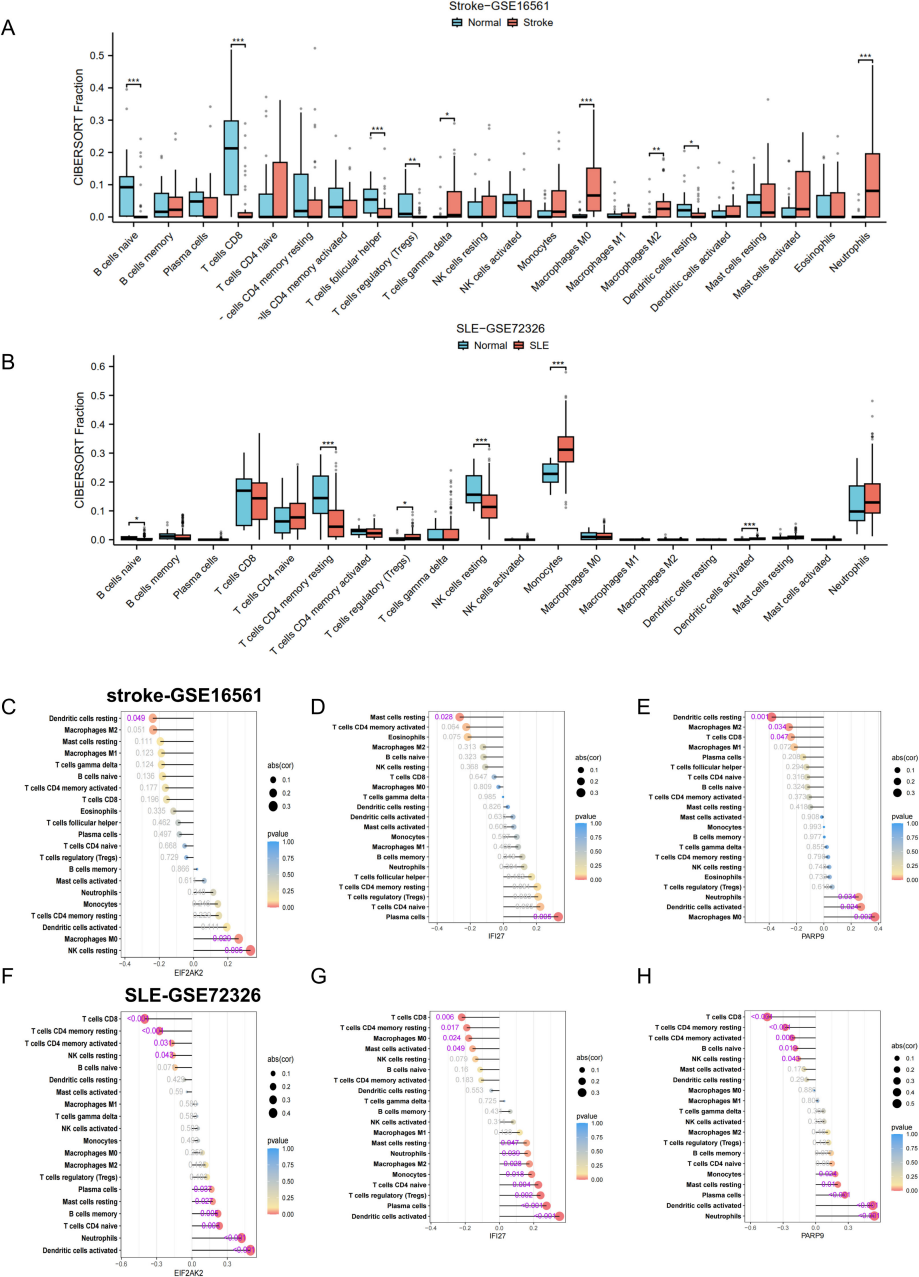
Correlation of hub genes and immune cell infiltration in stroke and SLE. **(A, B)** Boxplots showing the pattern of immune cell infiltration in the stroke-GSE16561 cohort and the SLE-GSE72326 cohort. **(C-H)** Lollipop plots showing the correlation between hub genes and immune cells. *p < 0.05, **p < 0.01, ***p < 0.001.

Following our enrichment analysis that highlighted the significance of the immune system in the progression of both conditions, we proceeded to investigate if the CIBERSORT method could discern distinct immune infiltration patterns, utilizing data on 22 distinct immune cell types. We commenced by examining the datasets for stroke and SLE. The differential expression analysis revealed congruent patterns of gene expression differences in both conditions when contrasted with controls. Notably, there was an increased presence of monocytes and neutrophils in stroke and SLE patients’ samples as opposed to those from healthy individuals, while CD8 T cells infiltration was significantly lower (**Figures 5A, B**), suggesting that both stroke and SLE seem to experience disruptions in immune regulation and exhibit inflammatory reactions.

To deepen our comprehension of the functional significance of three pivotal genes in immune cell infiltration, we performed a Spearman correlation analysis to examine the link between the expression levels of these genes and the presence of immune cells. The findings revealed significant correlations between the genes EIF2AK2, PARP9, and IFI27 and a range of immune cell populations. The correlation analysis indicated an inverse relationship between the genes EIF2AK2, PARP9, and IFI27 and the presence of M2 macrophages within the stroke dataset (**Figures 5C–E**). Notably, EIF2AK2 expression demonstrated the strongest positive association with resting NK cells, whereas it showed the most pronounced negative association with resting dendritic cells (**Figure 5C**). In terms of IFI27, there was a significant positive correlation with plasma cells and a marked negative correlation with resting mast cells (**Figure 5D**). In the SLE dataset, EIF2AK2, PARP9, and IFI27 had a significant positive correlation with neutrophil levels and a negative correlation with both CD8 T cells and resting NK cells (**Figures 5F–H**). Given that the majority of these pivotal genes exhibit elevated expression in disease states, such as stroke or SLE, this implies an increased neutrophil enrichment in these conditions. The data suggest that these signature genes might participate in the infiltration of immune cells within the blood immune milieu of stroke patients who have suffered SLE, thereby impacting disease progression.

### 3.7 Enrichment analysis of characteristic genes

**Figure 6:**
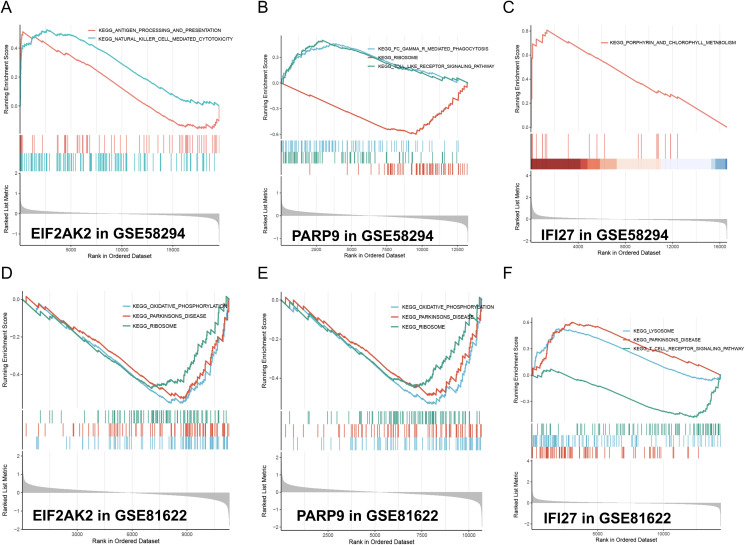
GSEA analysis of model feature genes in stroke and SLE. **(A-C)** GSEA analysis illustrates the enrichment of gene sets associated with EIF2AK2, PARP9, and IFI27 in the stroke-GSE58294 dataset. The running enrichment scores (RES) are plotted against the rank in ordered dataset. The blue and red lines represent different gene sets. The bar graphs at the bottom show the position of individual genes within the ranked list. **(D-F)** GSEA analysis similarly shows the enrichment of gene sets associated with EIF2AK2, PARP9, and IFI27 in the SLE-GSE81622 dataset, maintaining the same format as the stroke dataset analysis.

To gain deeper insights into the pathways linked to the signature genes, we conducted single-gene GSEA analysis. The GSEA findings revealed that among the high and low expression groups defined by the characteristic gene samples, several key signaling pathways were significantly enriched. These included the oxidative phosphorylation, lysosome, and phagocytosis pathways (enrichment score > 0.5, p < 0.05, **Figures 6A–F**), which play crucial roles in cellular physiology and pathology. The oxidative phosphorylation pathway, a critical process for ATP production via the electron transport chain and chemiosmosis across the mitochondrial inner membrane, was notably downregulated in the context of EIF2AK2 and PARP9. The lysosome pathway, involving the intracellular organelles that contain hydrolytic enzymes for the degradation of cellular and extracellular macromolecules, was upregulated in the presence of IFI27, indicating its role in autophagy and material recycling. Phagocytosis pathway, a vital component of the immune response where immune cells internalize and digest pathogens or cellular debris, was enhanced in the condition of PARP9, highlighting its importance in host defense. Additional, the natural killer cell-mediated cytotoxicity pathway, which involves the recognition and elimination of virus-infected cells or tumor cells by natural killer cells through the release of cytotoxic granules, was upregulated in the context of EIF2AK2, suggesting a potential role in immune surveillance and tumor control. These findings provide valuable insights into the molecular mechanisms underlying these pathways and their implications in disease states.

### 3.8 Validation of hub genes

**Figure 7:**
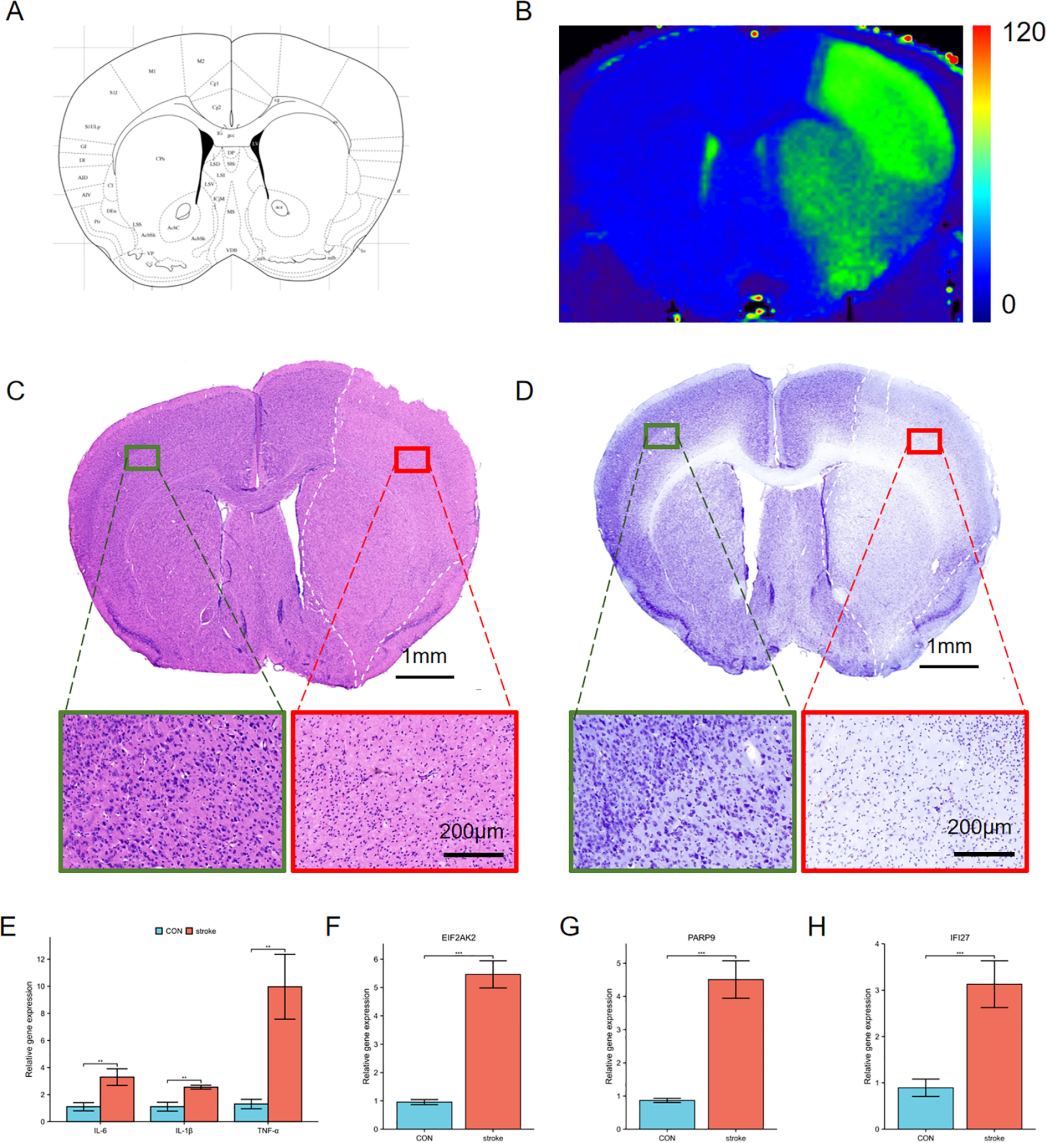
Characterization of ischemic brain injury and changes of hub genes in the stroke model. **(A)** Schematic representation of a coronal brain section from a mouse brain atlas. **(B)** T2 mapping derived from 9.4T MRI data. The right hemisphere exhibits higher T2 values (green area), indicative of edema compared to the left hemisphere. **(C, D)** H&E **(C)** and Nissl staining **(D)** of the ischemic cerebral cortex. Green squares indicate normal tissue, red squares denote ischemic regions, and white dashed lines outline edematous areas. Insets show higher magnification views of these regions. **(E)** The expression of proinflammatory cytokines IL-6, IL-1β and TNFα in the ischemic cerebral cortex. **(F-H)** The mRNA expression of EIF2AK2, PARP9 and IFI27 in the ischemic cerebral cortex. **p < 0.01, ***p < 0.001.

MRI T2 mapping was performed on MCAO mice, focusing on the region depicted in **Figure 7A**. The results revealed a significant increase in T2 values in the ischemic hemisphere (**Figure 7B**), indicative of pronounced edema in the affected brain tissue. Histological examination via H&E staining further elucidated the impact of ischemia. Notably, the ischemic area exhibited marked cellular shrinkage, as observed in **Figure 7C**, where the green square highlights a region of normal tissue for comparison, and the red square denotes the ischemic core with evident cellular damage. Additionally, Nissl staining provided insights into the neuronal integrity post-MCAO. The staining outcomes, shown in **Figure 7D**, underscored the severe damage inflicted upon neurons in the ischemic region. The qPCR analysis showed that the mRNA levels of IL-6, IL-1β and TNF-α were increased in the stroke area (**Figure 7E**). We examined the mRNA expression of the hub genes in the stroke area. The results showed that the expression of EIF2AK2, PARP 9 and IFI 27 was consistent with our DEGs analysis, and the gene expression was increased in stroke mice compared with non-infarcted area (**Figures 7F–H**). Therefore, we validated EIF2AK2, PARP 9 and IFI 27 as hub genes in the progression of stroke.

## 4 Discussion

SLE is notably associated with a heightened risk of stroke, affecting both ischemic and hemorrhagic subtypes (25). Patients with SLE experience a higher incidence of cerebrovascular diseases compared to the general population, with mortality rates from cerebrovascular events notably elevated. Neuropsychiatric systemic lupus erythematosus (NPSLE) is common among SLE patients, with cerebrovascular diseases being prevalent manifestations (26). The presence of antiphospholipid antibodies (aPL) correlates strongly with NPSLE symptoms, including headaches and seizures, while various pathological changes, such as microvascular damage and large vessel vasculitis, have been documented in SLE patients (26). Therefore, enhancing surveillance and management of cerebrovascular diseases is crucial in this patient population.

In our study, we identified 65 overlapping upregulated DEGs in datasets related to stroke and SLE. Through WGCNA, we pinpointed significantly correlated modules, ultimately narrowing our focus to 69 candidate driver genes. Enrichment analysis using GO and KEGG highlighted the upregulation of pathways relevant to cytokine-cytokine receptor interactions and immune response regulation, underscoring the importance of immune system dysregulation in the pathogenesis of both stroke and SLE.

We further explored the role of neutrophils in SLE, which release neutrophil extracellular traps (NETs) containing self-DNA-peptide complexes, activating plasmacytoid dendritic cells (pDCs) and promoting autoantibody production (27, 28). This process exacerbates inflammation by elevating interferon-alpha levels. During stroke, the immune system’s activation is multifaceted, involving both innate and adaptive immunity (12). The innate immune response contributes to early ischemic damage through the release of inflammatory mediators and activation of microglia, which produce cytokines and reactive species that can either promote or resolve inflammation.

Using Cytoscape software, we constructed a PPI network based on the 69 common driver genes to identify hub genes, ultimately selecting 10 as candidate diagnostic markers. Machine learning technique, LASSO regression analysis, was utilized to refine our search, leading to the identification of three genes, EIF2AK2, PARP9, and IFI27, which demonstrated strong diagnostic potential confirmed by ROC curves in both stroke and SLE cohorts. Importantly, all three genes exhibited consistent upregulation in both conditions compared to controls.

EIF2AK2, belonging to the family of eukaryotic initiation factor 2-alpha kinases, plays a role in modulating immune reactions and the regulation of protein synthesis under stressful conditions (29–31). Variants in EIF2AK2 have been linked to neurodevelopmental and neurodegenerative disorders, and its inhibition has shown promise in improving cognitive deficits in Alzheimer’s disease models, suggesting a potential therapeutic avenue (32, 33). Moreover, EIF2AK2 is essential for CD4 T cell survival and function, with its absence exacerbating autoimmune conditions associated with Th17 cells (34). PARP9 is involved in DNA damage repair, transcription regulation, and immune responses, particularly in the enhancement of interferon-mediated antiviral defenses (35, 36). Elevated PARP9 expression is associated with poor prognosis in glioblastoma (37), indicating its role in central nervous system diseases, where excessive DNA damage response may contribute to neuronal apoptosis following a stroke. IFI27, an interferon-inducible gene, plays a pivotal role in regulating cellular responses to interferons, and its closely related family member IFI27L2A is upregulated in microglia after a stroke (38). This IFI27 gene may influence Th1 cell development and immune responses, while its regulation could impact inflammation and recovery processes (39). Notably, IL-27, associated with IFI27, has been linked to the inhibition of Th17 cells, which are critical in SLE pathogenesis (40). Together, these hub genes (EIF2AK2, PARP9, and IFI27) are integral to the regulation of autoimmune and inflammatory responses, representing potential biomarkers for SLE with concomitant stroke. Their involvement in both pathologies highlights their significance as therapeutic targets and diagnostic indicators. Future studies should delve deeper into their functional roles and explore clinical applications.

In assessing immune cell infiltration patterns in stroke and SLE using CIBERSORT, we found similarities, notably a decrease in monocytes and resting NK cells. Previous findings indicate that SLE patients have reduced NK cell counts and cytotoxicity, possibly due to IFN-α-mediated cell death (41). This deficiency correlates with the progression of lupus nephritis. Similarly, NK cells can mitigate brain inflammation and assist in the clearance of pathological proteins, but they are also functionally impaired in stroke, which compounds inflammation through increased cytokine release (42, 43). Furthermore, macrophage polarization and activation are critical in both stroke and SLE pathogenesis, as they secrete pro-inflammatory factors that contribute to blood-brain barrier disruption and neuronal damage in ischemic stroke (44, 45). This highlights the crucial role of immune dysregulation and inflammatory responses in these conditions.

Validation of the expression of the three core genes at the animal level in a MACO model indicated significant increases in inflammatory factors and core gene expression. This study marks the first exploration of hub genes in the context of stroke and SLE, suggesting EIF2AK2, PARP9, and IFI27 as potential biomarkers for further investigation into the mechanistic underpinnings of their comorbidity. While our findings offer significant insights, limitations exist, particularly the reliance on bioinformatics without experimental validation. Future investigations should include experimental studies of these genes in relevant disease models and larger clinical cohorts to fully understand their roles as biomarkers. To enhance the precision of immune cell infiltration data, subsequent experiments will incorporate advanced techniques such as flow cytometry and single-cell RNA sequencing. These methods offer high-resolution insights into immune cell heterogeneity and dynamics, which are essential for unraveling the complex immunological landscapes in stroke and SLE. By integrating these techniques, we aim to provide a more detailed characterization of immune cell subsets and their roles in disease pathogenesis.

## 5 Conclusion

In this study, we found that the upregulation of inflammatory responses could represent a shared etiological pathway in both stroke and SLE. Furthermore, we have identified EIF2AK2, PARP9, and IFI27 as significant biomarkers for diagnostic purposes. Additionally, the analogous patterns of immune cell infiltration observed in stroke and SLE suggest potential avenues for therapeutic intervention.

## Data Availability

The datasets utilized in the study can be accessed through the Gene Expression Omnibus (GEO, https://www.ncbi.nlm.nih.gov/geo/): GSE16561, GSE58294, GSE72326, and GSE81622.

## Supplementary material

Supplementary Figure 1 Venn plots of crossover candidate drugs for the three hub genes.
