# Supplementary figures and images for: Shared circulating diagnostic biomarkers and molecular mechanisms in ischemic stroke and systemic lupus erythematosus
Source: Front Immunol. 2025 Apr 17;16:1565379. doi: 10.3389/fimmu.2025.1565379 (PMC12043496; doi:10.3389/fimmu.2025.1565379)

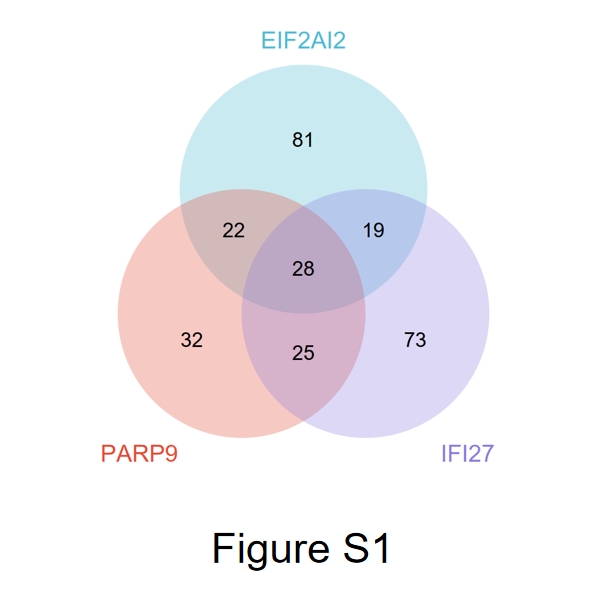

Supplement: Supplementary Figure 1 — Venn plots of crossover candidate drugs for the three hub genes. [file Image1.jpeg]
